# Editorial: Crosstalk within neurovascular unit: endothelial implications for the development and function of brain

**DOI:** 10.3389/fncel.2024.1440772

**Published:** 2024-06-10

**Authors:** Kunwei Wu, Shumin Gong, Xiang-Ping Chu

**Affiliations:** ^1^Jiangsu Province Key Laboratory of Anesthesiology, Jiangsu Province Key Laboratory of Anesthesia and Analgesia Application, NMPA Key Laboratory for Research and Evaluation of Narcotic and Psychotropic Drugs, Xuzhou Medical University, Xuzhou, Jiangsu, China; ^2^Departments of Biomedical Sciences and Anesthesiology, School of Medicine University of Missouri-Kansas City, Kansas City, MO, United States

**Keywords:** blood-brain barrier, neurovascular development, endothelial cells, angiogenesis, stroke

The neurovascular unit (NVU) is a complex network of cells within the brain that includes neurons, astrocytes, pericytes, and endothelial cells. This unit plays a crucial role in maintaining brain homeostasis and regulating cerebral blood flow. Brain endothelial cells, the building blocks of blood vessels, not only provide structural support but also actively participate in the communication between neurons and the vasculature. This bidirectional interaction is pivotal for various processes, including neurogenesis, synaptogenesis, and the maintenance of blood-brain barrier (BBB) integrity (Paredes et al., 2018; Sweeney et al., 2019; Wu et al., 2019). The intricate relationship between neurons and the vasculature within the brain is essential for its proper development and functioning. This editorial introduces a captivating Research Topic that delves into the concept of crosstalk within the neurovascular unit, with a specific focus on the crucial role of endothelial cells.

Regarding these, we are pleased to present the collection of papers in this Research Topic, Crosstalk Within neurovascular unit: endothelial implications for the development and function of brain. This Research Topic comprises a total of four articles. Three of these articles present original research findings, while one offers a comprehensive review of the existing literature. We anticipate that the research presented in this special topic will not only enhance our understanding of the intricate crosstalk within the neurovascular unit but also pave the way for potential therapeutic interventions targeting endothelial cells for the treatment of various neurological disorders. We extend our gratitude to the authors for their valuable contributions and invite readers to explore the fascinating world of the neurovascular unit and the pivotal role of endothelial cells in brain development and function.

During fetal development, the formation of blood vessels within the brain is essential for providing oxygen and nutrients to support the growth and maturation of neurons. Prenatal alcohol exposure (PAE) has been shown to have detrimental effects on fetal development, particularly in the central nervous system (Riley et al., 2011). Therefore, it is imperative to investigate the impact of PAE on the “placenta-brain” axis, which plays a critical role in fetal brain angiogenesis and neurovascular development. The original research article by Sautreuil et al. focused on placental CD146, a protein involved in angiogenesis and vascular development. They explored the effects of PAE on CD146 expression and its downstream signaling components, unraveling its significance in both placental and neurovascular development. The findings emphasize how dysregulation of CD146 expression due to PAE can disrupt the delicate balance required for proper cortical vasculature formation.

Dysfunction of endothelial cells contribute to the development of edema, inflammation, and secondary injury cascades following spinal cord injury (SCI). Moreover, impaired endothelial cell migration, tube formation, and barrier function in the spinal cord microvasculature can hinder tissue repair and regeneration processes (Bartanusz et al., 2011). The original research article by Scholpa et al. investigated the effects of Lasmiditan, a selective 5-HT_1F_ receptor agonist, on endothelial cell function and mitochondrial biogenesis (MB) in a mouse model of SCI. The results show that Lasmiditan treatment induces MB, promotes vascular recovery, and improves locomotor function. In primary cerebral microvascular endothelial cells, Lasmiditan increases mitochondrial proteins and density, enhances endothelial cell migration and tube formation, and improves barrier function. The article sheds light on the underlying mechanisms driving these beneficial effects. It reveals that the activation of the 5-HT_1F_ receptor leads to the activation of intracellular signaling pathways, including AMPK and PGC-1α, known regulators of MB. This mechanistic insight provides a deeper understanding of the molecular pathways involved in the observed mitochondrial enhancements and highlights potential targets for future therapeutic interventions.

The BBB is a crucial physiological barrier that tightly regulates the exchange of substances between the bloodstream and the central nervous system. Enhancing BBB penetration and increasing its permeability are key strategies to improve the transport of therapeutic agents across the BBB and enhance their efficacy in treating brain disorders. The original research article by Gong et al. investigated the effects of electroacupuncture applied to the trigeminal nerve on the modulation of BBB permeability and neuronal excitability. The researchers provide compelling evidence that electroacupuncture triggers blood-brain barrier opening mediated by N-methyl-D-aspartate (NMDA) receptors and subsequently induces changes in neuronal excitatory responses. The ability to transiently open the BBB through electroacupuncture could pave the way for improved drug delivery to the CNS, thereby enhancing the efficacy of therapeutic interventions for neurological disorders.

The BBB serves as a protective barrier, tightly regulating the exchange of molecules between the blood and the brain. Disruption of BBB integrity during ischemic stroke contributes to the entry of harmful substances and inflammatory mediators into the brain, exacerbating tissue damage. Multiple studies investigating this process highlight the significant role of microRNAs in the disruption of BBB integrity (Ma et al., 2020). The original review by Payne et al. discussed the involvement of microRNA-34a in ischemic stroke, specifically its interaction with mitochondrial genes in brain endothelial cells, the effect on mitochondrial function, and lastly its regulatory role in BBB permeability. The identification of microRNA-34a as a key player in BBB permeability and mitochondrial function provides a springboard for the development of effective strategies to combat ischemic stroke. Targeting microRNA-34a or its downstream signaling pathways may offer new opportunities for preserving BBB integrity and mitigating mitochondrial dysfunction.

## Author contributions

KW: Writing – original draft, Writing – review & editing. SG: Writing – original draft, Writing – review & editing. X-PC: Writing – review & editing.
